# Dosage in Chloroform Administration

**Published:** 1901-03-02

**Authors:** 


### DOSAGE IN CHLOROFORM ADMINISTRATION.

On the appearance of the report of the Anæsthetics Committee of the British Medical Association, we pointed out how very indefinite was its teaching, and how serious was the omission of any statement as to the best method of administering chloroform. We are glad to see that Dr. Augustus Waller, F.R.S., has taken up this matter on the same lines, and has published a stringent criticism of the finding of the committee, dealing in the first place with the want of definiteness in many of its conclusions, and then more especially with the absence of all guidance as to whether, in the administration of chloroform, the quantity given should be measured or not. "We are anxious above all," he says, "to know whether a method of administration elaborated during the last twenty-five years from a crude device due to Junker, and crudely applied to animals in every physiological laboratory, is or is not what it has been repeatedly claimed to be, on the strength of data published . . . over the names of experienced medical practitioners, namely, a volumetric method for the relatively 'safe' administration of anæsthetics." There can be no doubt, we think, that whatever may be the case among experts in anæsthesia, who by practice get to know pretty well what they are doing, among general practitioners the great question is whether to measure or not to measure, and on this point the committee, according to Dr. Waller, where it is not dumb is misleading. Dr. Waller sums up the report of the committee as follows:—"What has the ordinary administrator of anæsthetics learned from this clinical inquiry? That chloroform is more dangerous than ether, but that as regards methods of administration, rate of use methods of restoration, clinical evidence has not warranted any conclusion. That special circumstances may render the use of some anæsthetic, or combination of anæsthetics, both safer and easier than that of ether or nitrous oxide, but that no indications are derivable from the present inquiry as to the nature of the anæsthetic, or combination of anæsthetics, that it may be desirable to employ, with the exception of the A.C.E. mixture, which holds an intermediate place between chloroform and ether, but is more dangerous to males than females. And the most important clinical conclusion is that by far the most important factor in the safe administration of anæsthetics is the experience of the administrator, and that in many cases the anæsthetisation is of such importance and gravity that it is absolutely essential that an anæsthetist of large experience should conduct the administration;" and, as he truly adds, the last condition is not always easy to fulfil. Clinical experience has done its best, and this is as far as it takes us; and Dr. Waller urges that certain definite steps ought now to be taken to increase our knowledge on the subject, and among these he places first an experimental examination of the statement that by proper application of a volumetric method anæsthesia can be certainly effected of any required degree. The matter is one of great importance, and, whatever anæsthesia specialists may say on the subject, most practitioners, who are in the habit of laying considerable stress on the question of dosage in regard to every other drug, will agree that, if it is possible to measure one's chloroform as one goes on, it is very desirable that it should be done.

